# Long-term outcome of delirium in critically ill patients

**DOI:** 10.1186/cc12338

**Published:** 2013-03-19

**Authors:** A Wolters, D Van Dijk, O Cremer, D De Lange, A Slooter

**Affiliations:** 1 UMC Utrecht, the Netherlands

## Introduction

In ICU patients, little research has been performed on the relationship between delirium and long-term outcome, including health-related quality of life (HRQoL), cognitive functioning and mortality. In addition, results seem to be inconsistent. Furthermore, in studies that reported increased mortality in delirious patients, no proper adjustments were made for severity of illness during ICU admission. This study was conducted to investigate the association between ICU delirium and long-term HRQoL, cognitive functioning and mortality. The hypothesis was that delirious patients have worse long-term outcome in comparison with nondelirious patients.

## Methods

A prospective observational cohort study was conducted. A median of 12 months after ICU discharge, questionnaires were sent to all survivors. HRQoL and cognitive functioning were measured with the EuroQol-6D. Age, gender and severity of illness were considered relevant covariates. Severity of illness was estimated using the APACHE IV score and the maximal SOFA score during admission. HRQoL was investigated with linear regression analysis, cognitive functioning using logistic regression and mortality with Cox regression analysis.

## Results

The patient population consisted of 690 patients admitted to the ICU, subdivided into delirious (*n *= 257) and nondelirious patients (*n *= 433). During follow-up, 181 (26%) patients died. The response rate of the questionnaire was 70.6%. After adjusting for the predefined covariates, delirium was significantly associated with a lower HRQoL (adjusted β: -0.137; 95% CI = -0.140 to -0.005) and more mild and severe cognitive impairment (adjusted odds ratio: respectively: 2.3; 95% CI = 1.3 to 4.2 and 5.8; 95% CI = 1.3 to 15.2). No significant association between delirium and long-term mortality was found (adjusted hazard ratio: 1.0; 95% CI = 0.7 to1.4).

## Conclusion

Delirium during ICU admission was associated with lower HRQoL and worse cognitive functioning, 1 year after discharge. Furthermore, delirium on the ICU was not associated with long-term mortality after adjusting for relevant covariates, including severity of illness during ICU admission.

